# Deoxycholic Acid Modulates Cell-Junction Gene Expression and Increases Intestinal Barrier Dysfunction

**DOI:** 10.3390/molecules27030723

**Published:** 2022-01-22

**Authors:** Huawei Zeng, Bryan D. Safratowich, Wen-Hsing Cheng, Kate J. Larson, Mary Briske-Anderson

**Affiliations:** 1 United States Department of Agriculture, Agricultural Research Service, Grand Forks Human Nutrition Research Center, Grand Forks, ND 58203, USA; bryan.safratowich@usda.gov (B.D.S.); kate.larson@usda.gov (K.J.L.); mary.briske-anderson@usda.gov (M.B.-A.); 2 Department of Food Science, Nutrition and Health Promotion, Mississippi State University, Starkville, MS 39762, USA; wcheng@fsnhp.msstate.edu

**Keywords:** cell junction, bile acid, deoxycholic acid, gene expression, intestinal permeability

## Abstract

Diet-related obesity is associated with increased intestinal hyperpermeability. High dietary fat intake causes an increase in colonic bile acids (BAs), particularly deoxycholic acid (DCA). We hypothesize that DCA modulates the gene expression of multiple cell junction pathways and increases intestinal permeability. With a human Caco-2 cell intestinal model, we used cell proliferation, PCR array, biochemical, and immunofluorescent assays to examine the impact of DCA on the integrity of the intestinal barrier and gene expression. The Caco-2 cells were grown in monolayers and challenged with DCA at physiological, sub-mM, concentrations. DCA increased transcellular and paracellular permeability (>20%). Similarly, DCA increased intracellular reactive oxidative species production (>100%) and accompanied a decrease (>40%) in extracellular signal-regulated kinase 1/2 (ERK1/2) signaling pathways. Moreover, the mRNA levels of 23 genes related to the epithelial barrier (tight junction, focal adhesion, gap junction, and adherens junction pathways) were decreased (>40%) in (0.25 mM) DCA-treated Caco-2 cells compared to untreated cells. Finally, we demonstrated that DCA decreased (>58%) the protein content of occludin present at the cellular tight junctions and the nucleus of epithelial cells. Collectively, DCA decreases the gene expression of multiple pathways related to cell junctions and increases permeability in a human intestinal barrier model.

## 1. Introduction

The increasing worldwide incidence of colon cancer is linked to obesity and the intake of saturated fatty acids [1,2,3,4]. A high intake of fat induces the release of bile acids from the gall bladder into the small intestine. Then, bacteria in the colonic lumen convert conjugated and hydrophilic bile acids to unconjugated, hydrophobic bile acids known as secondary bile acid such as deoxycholic acid (DCA) and lithocholic acid [5]. The total bile acid concentration can reach approximately 1 mM in the colon after the consumption of high-fat meals with DCA (≈0.6 mM) being the primary secondary bile acid in humans [5,6,7]. Secondary bile acids such as DCA are implicated in the promotion of intestinal permeation and colon carcinogenesis [8].

The intestinal mucosal barrier serves as an important defense system by providing physical and functional barriers against invading pathogens [9]. Intestinal barrier permeation is achieved either by passage through the epithelial cells (the transcellular pathway) or through the intercellular space between adjacent epithelial cells (the paracellular pathway) [10]. Impaired epithelial barrier function (e.g., permeation) is implicated in the etiology of several diseases including colonic inflammation and cancer [11]. Various environmental factors such as high-fat diets impair the intestinal barrier including intestinal epithelial damage, mucus layer changes, and dysbiosis. These impairments result in a variety of intestinal diseases such as colon cancer [12]. Although DCA is considered a cancer-promoting agent in the colon, most previous studies on DCA and epithelial-barrier impairment have been confined to the tight junction pathway [5,12,13]. There are scant data examining the impact of DCA on other cell-junction pathways. In this study, we hypothesize that DCA modulates the gene expression of multiple cell-junction pathways and impairs intestinal integrity in the well-established Caco-2 cell model. With the combination of molecular, cellular, and bioinformatic approaches, we challenged the intestinal model with DCA at physiological, sub-mM, concentrations.

## 2. Results

### 2.1. Effects of DCA on Permeability in Caco-2 Monolayer

**Figure 1:**
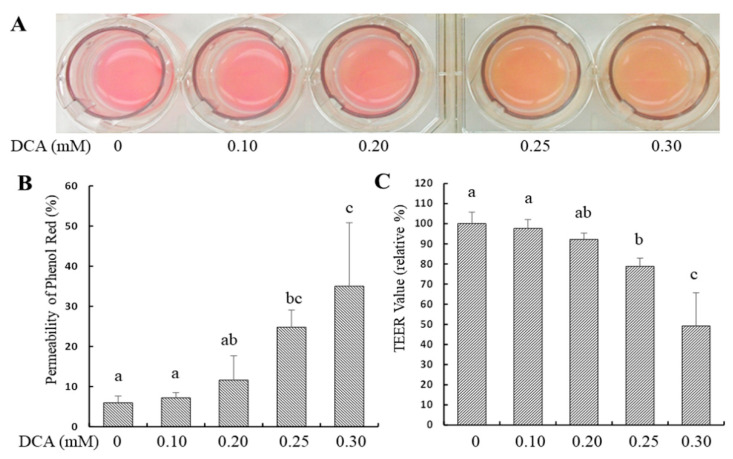
Effect of DCA treatment on intestinal barrier function in Caco-2 cell monolayers. (**A**) Representative phenol red photos of membrane inserts (apical side) that hung inside the chambers of six-well plates for 15 h; (**B**) DCA increased the phenol red level (%) in the membrane inserts’ basal side due to an increase in cell monolayers’ permeability; (**C**) DCA induced TEER decrement (%) and increased cell monolayers’ permeability. Values are means ± SDs, n = 4 independent experiments. Means without sharing a common letter differ, *p* < 0.05. The lower TEER value (relative%) means the higher permeability.

The permeability was increased by 19% and 29% in Caco-2 monolayers treated with 0.25 and 0.3 mM DCA via phenol red measurement compared to that of untreated cells, respectively (Figure 1A,B). Similarly, the permeability was increased by 21% and 51% in Caco-2 monolayers treated with 0.25 and 0.3 mM DCA via transepithelial electrical resistance (TEER) measurement, respectively (Figure 1A,C).

### 2.2. Effects of DCA on Intracellular Reactive Oxygen Species (ROS) Production

**Figure 2:**
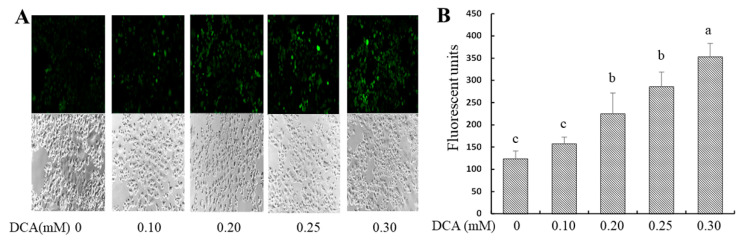
Effect of DCA treatment on ROS levels in Caco-2 cell monolayers stained by 2′,7′-dichlorofluorescein diacetate (DCFH-DA) probe for 15 min. (**A**) A representative intracellular ROS staining fluorescent image vs. brightfield image control; (**B**) the intracellular ROS staining fluorescent units. The area of intracellular ROS staining fluorescent signal was detected by a fluorescent microscope and quantitated by the Image Pro Plus Version 9.1 software. Values are means ± SDs, n = 4 independent experiments. Means without sharing a common letter differ, *p* < 0.05.

The intracellular 2′,7′-dichlorofluorescein (DCF) concentration was used as an indicator of ROS content. As noted, cellular oxidative stress impairs gut permeability [14], and intracellular ROS promotes gut leakage [15]. ROS content was increased by 80%, 130%, and 190% in Caco-2 cells treated with 0.2, 0.25, or 0.3 mM DCA, respectively, when compared to untreated cells (Figure 2A,B).

### 2.3. Effects of DCA on ERK1/2 and c-Myc Signaling Pathways and Cell Proliferation

**Figure 3:**
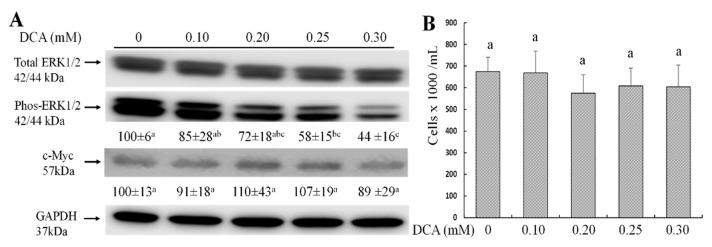
Effect of DCA on ERK1/2 and c-Myc signaling proteins and cell proliferation in Caco-2 cells for 15 h. (**A**) Representative Western blotting images were displayed, and band intensity was normalized to GAPDH protein. Phospho-ERK1/2 was further normalized to total ERK1/2; (**B**) Effect of DCA on the proliferation of Caco-2 cells. Values are means ± SDs, n = 4 independent experiments. Means without sharing a common letter differ, *p* < 0.05.

Cellular ROS modifies ERK1/2 and c-Myc signaling pathways, which play a crucial role in intestinal epithelial cell proliferation and permeability [16,17,18]. Although the protein level of c-Myc and cell proliferation did not differ, phosphorylated ERK1/2 were decreased by 42% and 56% in Caco-2 cells treated with 0.25 or 0.3 mM DCA when compared to untreated cells (Figure 3A,B). 

### 2.4. Effects of DCA on Cell Junction Gene Expression

**Figure 4:**
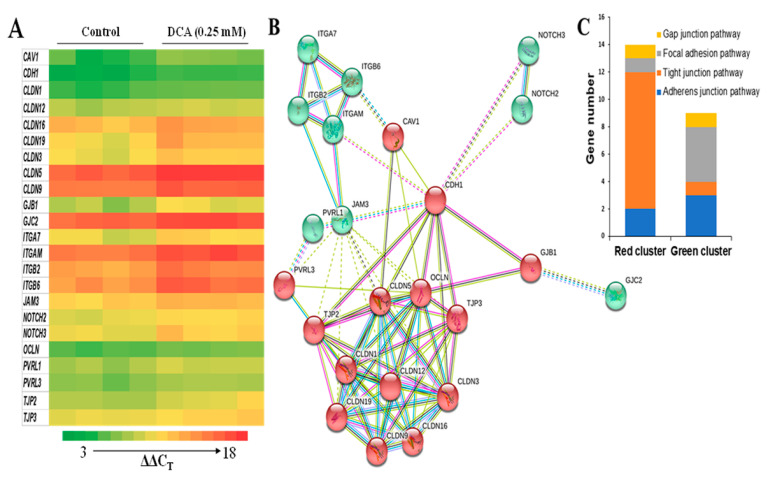
The functional analysis of 23 highly differentially expressed (>40% decrease) genes in Caco-2 cells with or without DCA treatment (0.25 mM) for 15 h. (**A**) A heat map of 23 highly differentially expressed (>40% decrease) genes in Caco-2 cells with or without DCA treatment (0.25 mM) for 15 h. The ΔΔC_T_ values are color-coded, and each column represents a set of separate, independent experiments (n = 4 independent experiments, the higher ∆∆C_T_ values and the lower gene expression). Means of all 23 genes’ ∆∆C_T_ values differ (*p* < 0.05; FDR-corrected Student’s *t* tests). (**B**) Two major protein–protein interaction clusters (green and red filled circles) in these 23 genes: 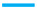 from curated databases; 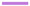 experimentally determined; 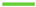 textmining; 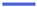 protein homology; 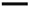 co-expression; 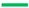 gene neighborhood; 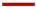 gene fusions. (**C**) The number of genes involved in tight junction vs. other cell junction combined (focal adhesion, gap junction, and adherens junction) pathways in these 23 genes.

To identify the major genes involved in the permeability in Caco-2 monolayers, we performed a PCR cell junction gene array that detects the mRNA levels of 84 key cell junction genes. To reduce the bystander gene effect, cells treated with DCA at 0.25 mM were chosen, because this was the lowest concentration to induce hyperpermeability (Figure 1). The mRNA content of 23 genes were decreased at least 40% in DCA-treated Caco-2 cells when compared to untreated cells (Figure 4A, Table 1). These down-regulated genes are involved in focal adhesion, tight junction, gap junction, and adherens junction pathways [19,20]. Protein–protein interaction analyses indicated that these 23 cell junction genes were concentrated into two clusters: (1) tight junction pathways and (2) focal adhesion, gap junction, and adherens junction pathways (Figure 4B,C).

### 2.5. Effects of DCA on Occludin Protein Level and Cellular Localization

**Figure 5:**
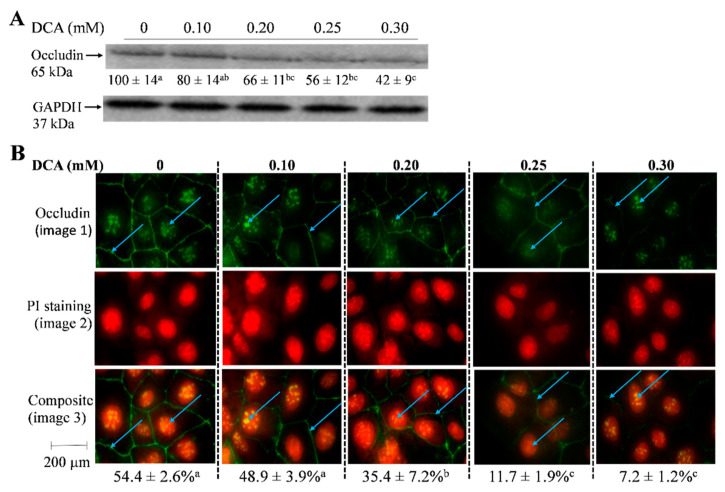
Effect of DCA treatment (0–0.3 mM) for 15 h on occludin protein expression and cellular localization in Caco-2 cells. (**A**) Values are means ± SDs, n = 4 independent studies. Means without Scheme 0. Representative Western blotting images were displayed, and band intensity was normalized to GAPDH; (**B**) Each concentration (a column) consists of three images: image 1, cells were labeled with anti-occludin antibody, followed by anti-Rabbit IgG (H + L), F(ab’)_2_ Fragment (Alexa Fluor^®^ 488 Conjugate) (green signals); image 2, cells were mounted by fluoroshield with PI as counter staining for overall cell morphology (red signals); image 3, the occludin protein image was superimposed on the respective overall cell morphology background image to generate the composite image (orange signals). Caco-2 cells with intense occludin expression around the tight junction and nucleus were indicated by blue arrows (1000× magnification). For composite images, the area of occludin protein level (green signals) in percentage was calculated in comparison with that of overall cell morphology (red signals). Values are means ± SDs, n = 4 independent experiments. The area of intracellular fluorescent signal was detected by a fluorescent microscope and Image Pro Plus Version 9.1 software. Means without sharing a common letter differ, *p* < 0.05.

As many differential genes occurred in the tight-junction pathway (Figure 4, Table 1), we then focused on occludin, which is a key component of tight-junction strands [21]. Consistent with a drop of mRNA level (Figure 4, Table 1), the protein content of occludin was decreased by 34% and 58%, respectively, in cells treated with 0.25 mM and 0.30 mM DCA when compared to untreated cells (Figure 5A). Furthermore, we found that occludin was located at both the cell membrane and nucleus. The ratio percentage (composite image) of occludin protein level versus overall cell background was decreased by 78% and 87%, respectively, in the cells treated with 0.25 mM and 0.30 mM DCA when compared to untreated cells (Figure 5B).

## 3. Discussion

The abundance of DCA is much higher than other secondary bile acid species (e.g., lithocholic acid) occurring in the feces [5,6,7]. Increasing concentrations of DCA in the colonic lumen may enhance gut permeation, causing an uncontrolled flux of antigens across the colonic epithelium that may challenge the immune system [12]. Colonic transit time in a healthy individual is highly variable, ranging from 14 to 80 h, depending on age, gender, and physical activities [22]. To avoid potential DCA cytotoxicity due to a long incubation time (e.g., 24 h or 48 h), 15 h treatment time in this report was chosen because it did not alter cell proliferation (viable cell count) (Figure 3B). In this study, we confirm the hypothesis that DCA modulates the gene expression in multiple cell junction pathways and increases intestinal permeability. We showed that the effect of DCA on TEER value was greater than of paracellular diffusion (phenol red) measurement at 0.3 mM (Figure 1). As TEER measurement detects both transcellular and paracellular permeation, these data suggest that DCA increased transcellular permeation. It has been reported that ROS can mediate DNA damage in a cell model and induce epithelial barrier dysfunction and colitis in a mouse model [15,23]. Consistent with these observations, DCA (>0.2 mM) also increased intracellular ROS levels (Figure 2). Along the same lines, ERK is a core-signaling pathway that allows the cell to respond to extracellular stimuli such as oxidative stress and regulates cell proliferation and intestinal barrier function [24,25]. Thus, we examined the effect of DCA on ERK1/2 signaling and found that DCA greatly reduced levels of phosphorylated ERK but not cell proliferation in Caco-2 cells (Figure 3A,B). This finding is consistent with that the activation of ERK is involved in the stabilization of tight junction integrity, and polyphenol extracts strengthen intestinal barrier function by activating ERK signaling in human intestinal cell models [25,26]. Therefore, that DCA inhibited ERK1/2 activation (Figure 3A), suggests, for the first time, that DCA destabilizes the tight junction integrity because of weak ERK signaling, and it subsequently induces epithelial barrier dysfunction. In line with the above cellular signaling, the c-Myc protein is a proto-oncogene product associated with oxidative stress, cell proliferation, and intestinal permeability [27,28]. Our data showed that c-Myc protein levels did not differ between DCA-treated cells and untreated cells (Figure 3A), suggesting that the c-Myc signaling pathway was not critically associated with intestinal permeability.

The uptake and transport of molecules in and between the epithelial cells are highly regulated through a junctional complex which includes focal adhesions, tight junction, gap junction, adherens junctions, and desmosomes [12,29]. While there are ample studies on tight junctions, scant data examine the effect of DCA on the gene expression of other types of cell junctions. We found that DCA reduced 23 (out of 84) junctional gene’s expression, which included not only tight junction genes but also focal adhesion, gap junction, and adherens junction genes (Figure 4, Table 1). The protein–protein interaction analysis showed that genes in the tight junction pathway were mainly in one cluster, while genes in the focal adhesion, gap junction, and adherens junction pathways were mainly in the other cluster (Figure 4). *CAV1*, *CDH1*, *GJB1*, *JAM3*, and *PVRL3* were bridge genes between these two gene-clusters (Figure 4, Table 1), indicating that these five genes might play a bridge role in the overall functional interaction for all 23 genes (Figure 4, Table 1). The above data suggest that the interaction between the focal adhesion, gap junction, and adherens junction pathways are greater when compared with the tight junction pathway, although all these cell junction genes may be functionally interactive. As DCA did not change the cell proliferation (Figure 3B), DCA-induced gut permeability (Figure 1) may not be directly related to the inhibition of epithelial cell proliferation in the intestinal barrier. Therefore, the inhibitory effect of DCA on multiple junction gene expression is a key mechanistic event underlying the increased colonic permeation in an epithelial cell barrier.

Another important aspect is the trafficking of junction proteins because a stimulation of junctional protein endocytosis can disrupt the epithelial barrier in response to inflammatory mediators [30]. Occludin is a tight junction protein that plays a prominent role in restricted paracellular transport [31,32], and ERK interacts directly with the C-terminal region of occludin, which is required for the stabilization of tight junction integrity [25]. In response to DCA treatment, the mRNA (Table 1) and protein levels of occludin were down-regulated (Figure 5). In addition to protein level, the epithelial barrier function of occludin may depend on its trafficking and subcellular localization [33,34]. Occludin is expressed in cellular locations without tight junctions such as the nucleus and the centrosome and regulates gene transcription (e.g., mRNA export to cytosol) and cell cycle progression in kidney, nerve, and other animal cell types [34,35,36]. Thus, we examined the effect of DCA on subcellular localization and found that DCA treatment decreased occludin expression in both tight junction and nucleus in the epithelial Caco-2 cells (Figure 5). These results lead us to speculate that increasing DCA concentrations may compromise both tight junction integrity in cell membrane and gene transcription efficacy in the nucleus of gut epithelial cells. While DCA concentrations can reach approximately 0.6 mM in the colon after the consumption of high fat meals in humans [5,6,7], a thick colonic mucus layer, generated by the cells of the colon’s wall, may reduce the lumenal DCA concentrations in contact with colonocytes. However, fiber-deprived diets are known to degrade this colonic mucus barrier, resulting in poor protection [37]. The interactions between diet and gut microbiome composition and bacterial metabolites regulate these mucus layer functionalities [37]. Due to the fact that <10% of Americans are getting the recommended daily amount of dietary fiber [38], it is conceivable that DCA concentrations at 0.1 to 0.3 mM and the effects on cell junction in this report are physiologically relevant. Given the potential effect of DCA-related gene transcription efficacy, it will be of great interest to examine the interplay between DCA treatment and the global transcription response, which would provide new insights into DCA and colon carcinogenesis. Future human studies are needed before extrapolating these data in clinical application.

**Figure 6:**
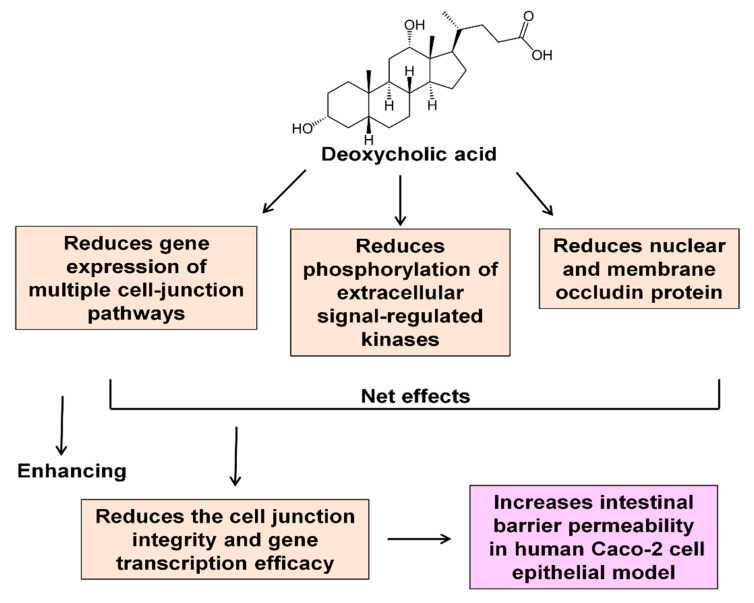
The summary of underlying molecular events related to DCA-induced intestinal barrier permeability in the human Caco-2 cell model.

Taken together, our data demonstrate, for the first time, that DCA treatment decreases the expression of genes in association with multiple cell-junction pathways including tight junction, focal adhesion, gap junction, and adherens junction. In addition, DCA may destabilize the tight junction integrity because of inhibiting ERK activation. Furthermore, DCA treatment decreases the protein level of occludin at both the tight junction and the nucleus, which may reduce gene transcription efficacy. These molecular events may represent the underlying mechanistic pathways that are responsible for DCA-induced intestinal hyperpermeability (Figure 6). Our results suggest that human fecal DCA concentration may serve as one of potential noninvasive analytes (biomarkers) for diet-related leaky gut in the context of obesity.

## 4. Materials and Methods

### 4.1. Cell Cultures and Cell Count

A human colon carcinoma Caco-2 cell line was obtained from the American Type Culture Collection (Manassas, VA, USA) at passage 17 and maintained in Dulbecco’s modified Eagle medium (DMEM; Invitrogen, CA, USA) with 10% fetal bovine serum (FBS; Atlanta Biologicals, Lawrenceville, GA, USA). Falcon polyethylene terephthalate high-density (PET-HD; 1.0 × 10^8^ pores/cm^2^, 0.45-μm pore size) membrane inserts were purchased from Becton-Dickinson Labware (Lincoln Park, NJ, USA). Cell proliferation assays (count and viability) were performed by using the trypan blue exclusion hemocytometer method [39]. Stock cells were passaged once weekly at approximately less than 90% confluency and incubated in a humidified chamber at 36.5 °C with 5% CO_2_. Cultures were tested and found to be mycoplasma-free [40]. Cells between passages 26 and 37 were seeded (58,000 cells/cm^2^) onto PET-HD membrane inserts that hung inside the chambers of six-well plates. Growth medium (1.5 mL; DMEM containing an additional 100 mL of fetal bovine serum (FBS), 4.5 mg glucose, 4 mmol glutamine, and 0.1 mmol of nonessential amino acids/L) was placed inside the insert (apical side) and 2.5 mL was placed in the chamber (basolateral side). The culture medium was changed at 3-day intervals until the cells had grown to confluence, differentiated, and begun to display characteristics of intestinal epithelium at day 9; then, the medium was changed every 2 days until the experiment was terminated at day 21 [41].

### 4.2. Detection of Intestinal Barrier Permeability in Caco-2 Monolayers

The integrity and permeability of the monolayers was determined by measuring the value of TEER using an Epithelial Voltohmmeter (World Precision Instrument; Haven, CT, USA) according to the manufacturer’s instructions. The TEER measurement, a continuous current passing through the cells, was used to assess both transcellular and paracellular permeation. The TEER values obtained in the absence of cells were considered as background-level measurements. All experiments were normalized to the TEER of the monolayer when it reached 350 Ω cm^2^ at 18–20 days after seeding cells on the Transwell inserts. For the phenol red assay, phenol red was included in the apical chamber to determine paracellular diffusion (permeation) through the Caco-2 cell monolayer at day 20. The percentage of phenol red transported into the basal chamber was calculated as previously described [42]. Briefly, 42 μM phenol red was included in the apical chamber. Aliquots of 100 μL were removed from the basal chamber after 15 h incubation at 37 °C and added with 1 M NaOH. The optical density at 558 nm of the basal chamber contents was measured to determine the passage of phenol red through the intercellular spaces [42].

### 4.3. Detection of Intracellular ROS Production

DCFH-DA (Molecular Probes, Eugene, OR, USA) is a nonfluorescent cell-permeable probe which is de-esterified intracellularly and rapidly oxidized to the highly fluorescent DCF by ROS [43]. For each assay, DCA-treated Caco-2 cells cultured on 24-well plates were pre-incubated with DCFH-DA (5 μM) solubilized in DMSO for 15 min at 37 °C and washed three times with PBS. The intracellular DCF was analyzed by a fluorescent microscope (Ex = 490 nm; Em = 510 nm). Image Pro Plus Version 9.1 software (Media Cybernetics, Inc., Rockville, MD, USA) was used for computerized quantification.

### 4.4. Human Cell Junction Gene Expression Array and Functional Gene Enrichment Analyses

The total RNA of Caco-2 cells was purified and treated with DNase using the RNeasy kit (Qiagen, Germantown, MD, USA). Quantitative and qualitative assessment of RNA samples were performed by NanoDrop spectrophotometry (Thermo Fisher, Inc., Waltham, MA, USA). The cDNA was synthesized by using 1 µg total RNA as the template with a reverse transcription Quantiscript reaction kit (Qiagen, Germantown, MD, USA). These cDNA products were run on the human cell junction PCR array (Cat#PAHS-213Z) (Qiagen, Germantown, MD, USA) following the manufacturer’s protocol. For functional gene enrichment analysis, the gene list was submitted and analyzed using the Kyoto Encyclopedia of Genes and Genomes (KEGG) and Search Tool for the Retrieval of Interacting Genes/Proteins (STRING) database search engine [44,45].

### 4.5. Immunofluorescent Staining

Caco-2 cells were seeded on microscopic slides (about 2 × 10^5^ cells per cell culture chamber slide) in DMEM media supplemented with 10% FBS under an atmosphere of 5% CO_2_ at 37 °C overnight. To observe the subcellular localization of occludin, the cells were pretreated with DCA for 15 h. After the treatment, cells were fixed using 4% paraformaldehyde for 15 min and then permeabilized with ice-cold 100% methanol for 10 min at −20 °C with PBS rinse for 5 min. Then, cells were blocked with 10% goat serum (Sigma Chemical Corporation, St. Louis, MO, USA) for 1 h, which was followed by incubation with an anti-occludin antibody (Thermo Fisher, Inc., Waltham, MA, USA) overnight at 4 °C. After washing with PBS, cells were incubated with anti-rabbit Immunoglobulin G (IgG, H, and L; F(ab’)2 Fragment; Alexa Fluor 488 Conjugate; green fluorescence (Cell Signaling Technology, Inc., Danvers, MA, USA) for 1 h at room temperature with propidium iodide (PI) (25 μg mL^−1^). Finally, fluoroshield with PI (Sigma Chemical Corporation, St. Louis, MO, USA), an aqueous mounting medium, was used for preserving fluorescence and producing a red fluorescence as counter stain for overall cell morphology. The fluorescence images and intensity quantification of at least 2000 cells per sample were analyzed by a Nikon E400 fluorescence microscope and Image Pro Plus version 9.1 (Media Cybernetics, Inc., Rockville, MD, USA).

### 4.6. Western Blotting Analysis

After DCA treatment, adherent cells were scraped and pooled with the detached cells in 5 mL media. Then, these cells were collected by centrifugation at 350× *g* for 10 min at 4 °C; 4 independent experiments were performed to collect the cells. The cell pellet was washed once in ice-cold PBS and lysed in an assay buffer (Cell Signaling Technology, Inc., Danvers, MA, USA) with 1 mM phenylmethylsulfonyl fluoride. After a brief sonication, the cell lysate was centrifuged at 14,000× *g* for 30 min at 4 °C. The supernatant was designated as whole cell protein extract and kept at −80 °C. The protein concentration was quantified by the Bradford dye-binding assay (Bio-Rad laboratories, Richmond, CA, USA). Protein extracts with equal amount (≈40 μg) were resolved over 4–20% Tris-glycine gradient gels under denaturing and reducing conditions and electroblotted onto PVDF membranes (Invitrogen, Carlsbad, CA, USA). Membrane blots were blocked in TBS with 0.05% Tween (*v*/*v*) and 5% (*w*/*v*) nonfat dry milk (BioRad, Hercules, CA, USA) at room temperature for 1 h. Membranes were probed with antibodies against occludin (Thermo Fisher, Inc., Waltham, MA, USA), c-Myc (Abcam, Cambridge, MA, USA), phospho-extracellular-regulated kinase 1/2 (ERK1/2), total ERK1/2, and glyceraldehyde-3-phosphate dehydrogenase (GAPDH) antibodies (Cell Signaling Technology, Inc., Danvers, MA, USA). Subsequently, the membranes were incubated with an anti-mouse/rabbit HRP-conjugated secondary antibody (1:3000; Cell Signaling Technology, Inc., Danvers, MA, USA) in blocking solution for 1 h at room temperature. Blots were washed, and proteins were incubated with an ECL plus kit (Thermo Fisher, Inc., Waltham, MA, USA) and imaged by the Molecular Dynamics Image-Quant system (Sunnyvale, CA, USA).

### 4.7. Statistical Analyses

The effect of DCA treatment on Caco-2 cell proliferation, permeability, ROS production, and tight junction protein level was analyzed by one-way ANOVA with Tukey contrast. The real-time PCR array data on DCA-treated Caco-2 cells were quantitated by using the comparative C_T_ method [46], and Caco-2 cells were used as controls for the real-time PCR (fold change = 1). For the amount of target as determined by real-time PCR, 1-, >1-, and <1-fold changes indicate no change, up-regulation, and down-regulation, respectively. The amount of target gene was normalized to endogenous references (GAPDH; ribosomal protein, large (RPLP0)). Subsequently, the data were analyzed using Student’s *t* tests for unequal variances with an applied false discovery rate (FDR) of 0.05, and only FDR-corrected *p*-values were reported. However, fold change (2^−ΔΔCT^) was reported in the text; 2^−ΔΔCT^ and ΔC_T_ values were used to calculated for standard deviation (SD) in DCA-treated (0.25 mM) and control (untreated) cells, respectively. All data are presented as means ± SD, and analyses were performed by using JMP V 12 software (SAS Institute, Inc., Cary, NC, USA).

## Figures and Tables

**Table 1:** Gene expression on cell junction in Caco-2 cells treated with deoxycholic acid (DCA).

Genes	Cluster	Pathways	Control (Untreated)	[0.25 mM] DCA
*CAV1*	Red	Focal adhesion	1.00 ± 0.17	0.35 ± 0.18 *
*CDH1*	Red	Adherens junction	1.00 ± 0.13	0.50 ± 0.20 **
*CLDN1*	Red	Tight junction	1.00 ± 0.07	0.55 ± 0.15 **
*CLDN12*	Red	Tight junction	1.00 ± 0.06	0.63 ± 0.21 *
*CLDN16*	Red	Tight junction	1.00 ± 0.04	0.48 ± 0.15*
*CLDN19*	Red	Tight junction	1.00 ± 0.07	0.26 ± 0.15 **
*CLDN3*	Red	Tight junction	1.00 ± 0.06	0.41 ± 0.14 **
*CLDN5*	Red	Tight junction	1.00 ± 0.03	0.35 ± 0.12 **
*CLDN9*	Red	Tight junction	1.00 ± 0.01	0.44 ± 0.10 **
*GJB1*	Red	Gap junction	1.00 ± 0.10	0.28 ± 0.11 **
*GJC2*	Green	Gap junction	1.00 ± 0.02	0.45 ± 0.08 **
*ITGA7*	Green	Focal adhesion	1.00 ± 0.08	0.40 ± 0.10 *
*ITGAM*	Green	Focal adhesion	1.00 ± 0.03	0.30 ± 0.14 **
*ITGB2*	Green	Focal adhesion	1.00 ± 0.03	0.34 ± 0.13 *
*ITGB6*	Green	Focal adhesion	1.00 ± 0.05	0.24 ± 0.11 **
*JAM3*	Green	Tight junction	1.00 ± 0.04	0.48 ± 0.16 **
*NOTCH2*	Green	Adherens junction	1.00 ± 0.04	0.48 ± 0.13 *
*NOTCH3*	Green	Adherens junction	1.00 ± 0.03	0.39 ± 0.16 *
*OCLN*	Red	Tight junction	1.00 ± 0.04	0.55 ± 0.06 *
*PVRL1*	Green	Adherens junction	1.00 ± 0.07	0.50 ± 0.22 **
*PVRL3*	Red	Adherens junction	1.00 ± 0.07	0.52 ± 0.16 **
*TJP2*	Red	Tight junction	1.00 ± 0.03	0.34 ± 0.18 *
*TJP3*	Red	Tight junction	1.00 ± 0.02	0.45 ± 0.09 *

Values are means ± SDs, n = 4 independent experiments. * *p* < 0.05 and ** *p* < 0.01, compared with untreated Caco-2 cells by using. FDR-corrected Student’s *t* test.

## Data Availability

The data presented in this study are available on request from the corresponding author.

## Sample Availability

Deoxycholic acids are available from the authors.

## Abbreviations

BA, bile acids; CAV1, Caveolin 1; CDH1, Cadherin 1; CLDN1, Claudin 1; CLDN12, Claudin 12; CLDN16, Claudin 16; CLDN19, Claudin 19; CLDN3, Claudin 3; CLDN5, Claudin 5; CLDN9, Claudin 9; DCA, deoxycholic acid; DCF, DCFH-DA, 2′,7′-dichlorofluorescein diacetate; ERK1/2, Extracellular Signal-Regulated Kinase 1/2; FDR, false discovery rate; GAPDH, Glyceraldehyde-3-Phosphate Dehydrogenase; GJB1, Gap junction protein beta 1; GJC2, Gap junction protein gamma 2; ITGA7, Integrin alpha 7; ITGAM, Integrin alpha M; ITGB2, Integrin beta 2; ITGB6, Integrin beta 6; JAM3, Junctional adhesion molecule 3; KEGG, Kyoto Encyclopedia of Genes and Genomes; NOTCH2, Notch homolog 2; NOTCH3, Notch homolog 3; OCLN, Occludin; PVRL1, Poliovirus receptor-related 1; PVRL3, Poliovirus receptor-related 3; ROS, reactive oxygen species; RPLP0, ribosomal protein, large; STRING, Search Tool for the Retrieval of Interacting Genes/Proteins; TEER, transepithelial electrical resistance; TJP2, 3, Tight junction protein 2, 3.
